# Autonomous Nucleic Acid and Protein Nanocomputing Agents Engineered to Operate in Living Cells

**DOI:** 10.1021/acsnano.4c13663

**Published:** 2025-01-06

**Authors:** Martin Panigaj, Tanaya Basu Roy, Elizabeth Skelly, Morgan R. Chandler, Jian Wang, Srinivasan Ekambaram, Kristin Bircsak, Nikolay V. Dokholyan, Kirill A. Afonin

**Affiliations:** Nanoscale Science Program, Department of Chemistry, University of North Carolina at Charlotte, Charlotte, North Carolina 28223, United States; Department of Pharmacology, Department of Biochemistry & Molecular Biology, Penn State College of Medicine, Hershey, Pennsylvania 17033, United States; Nanoscale Science Program, Department of Chemistry, University of North Carolina at Charlotte, Charlotte, North Carolina 28223, United States; MIMETAS US, INC, Gaithersburg, Maryland 20878, United States; Department of Pharmacology, Department of Biochemistry & Molecular Biology, Penn State College of Medicine, Hershey, Pennsylvania 17033, United States; Department of Pharmacology, Department of Biochemistry & Molecular Biology, Penn State College of Medicine, Hershey, Pennsylvania 17033, United States; MIMETAS US, INC, Gaithersburg, Maryland 20878, United States; Department of Pharmacology, Department of Biochemistry & Molecular Biology, Penn State College of Medicine, Hershey, Pennsylvania 17033, United States; Nanoscale Science Program, Department of Chemistry, University of North Carolina at Charlotte, Charlotte, North Carolina 28223, United States

**Keywords:** rational design, directed evolution, nucleic acid nanoparticles, nanocomputing agents, proteins

## Abstract

In recent years, the rapid development and employment of autonomous technology have been observed in many areas of human activity. Autonomous technology can readily adjust its function to environmental conditions and enable an efficient operation without human control. While applying the same concept to designing advanced biomolecular therapies would revolutionize nanomedicine, the design approaches to engineering biological nanocomputing agents for predefined operations within living cells remain a challenge. Autonomous nanocomputing agents made of nucleic acids and proteins are an appealing idea, and two decades of research has shown that the engineered agents act under real physical and biochemical constraints in a logical manner. Throughout all domains of life, nucleic acids and proteins perform a variety of vital functions, where the sequence-defined structures of these biopolymers either operate on their own or efficiently function together. This programmability and synergy inspire massive research efforts that utilize the versatility of nucleic and amino acids to encode functions and properties that otherwise do not exist in nature. This Perspective covers the key concepts used in the design and application of nanocomputing agents and discusses potential limitations and paths forward.

## Graphical Abstract

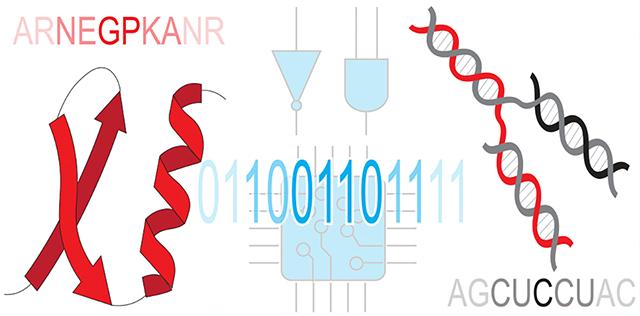

## INTRODUCTION

Over the past years, autonomous machines have revolutionized many human activities. With computational processing, cars, drones, robotic systems, and other emerging technologies operate with varying levels of independence from human control and are programmed to respond and adapt to changes in their surroundings.^1^ When handling biological systems, we have the advantage of working with natural processing units that have been evolving for billions of years and offer varying degrees of stepwise complexity. This allows for a straightforward approach to further development where we can modify these biological units to serve specific purposes, harnessing their inherent autonomous functions. For example, extensive research into transgene delivery by viruses has led to FDA approved therapies such as CAR T-cell therapy where T lymphocytes of patients are genetically engineered to attack cancer cells upon infusion.^2–6^ Oncolytic viruses exemplify another group of therapeutics that react to the environment and execute the predefined function accordingly, leading to cancer cell lysis.^7^ Symbiotic or parasitic human protozoa are attractive tools for the development of autonomous cell-based therapeutics, as documented by a recent study where *Toxoplasma gondii* was programmed to deliver multiple large (> 100 kDa) therapeutic proteins into neurons *in vivo*.^8,9^ Currently, genetically modified mosquitoes used for controlling vector-borne diseases are some of the most complex autonomous “machines” in biotechnology.^10^ However, creating autonomous biodevices that can operate on the cellular or subcellular level is still challenging. Modifying the functions of organisms or whole cells to function as nanobots requires extensive genetic reprogramming. Moreover, such “cellbots” may be prone to uncontrolled evolution. Viruses seem to be the ideal machinery for directed transport and execution of the designed program in targeted cells, yet the production of the necessary dose of recombinant viruses is not trivial, and genotoxic safety concerns and specificity may limit their widespread use. On the other hand, multicellular organisms may present several complications, hitherto unforeseen.

Adopting a bottom-up approach to develop programmable constructs enables the synthesis and assembly of biomaterials composed of nucleic acids and proteins with novel functionalities and implemented stimuli-responsive behaviors. By employing both natural and synthetic components, this method offers the opportunity to create smart and dynamic nanocomputing agents (NCAs) that exhibit a higher degree of adaptability and the freedom to evolve. These NCAs can self-assemble and interact in complex, programmable ways, mimicking, enhancing, or orchestrating a plethora of biological processes and allowing for the development of complex and responsive systems. Such an approach could transform biotechnology, including biomedicine, given the ability of these systems to dynamically adjust to changing environmental stimuli or specific biological signals, further expanding their potential applications. While nucleic acids and proteins are adaptable for NCA design, other essential biological components, such as lipids and carbohydrates, can also enhance and support NCA functionality. Despite lacking enzymatic activity, a lipid bilayer can act as a chemical circuit board to anchor NCAs, creating a “lipid nanotablet” platform. Within this platform, a nanoparticle logic gate detects molecules in the solution as inputs and initiates particle assembly or disassembly as outputs.^11^ Lipid membranes naturally compartmentalize and aid in the control of spatial communication. This concept inspires the design of structures, referred to by researchers as “nanoreactors”, which regulate interactions among lipid-bound molecular receptors across three primary dimensions: stoichiometric, spatial, and temporal.^12^ In general, lipid compartmentalization of signal perception, analysis, and response will be necessary in complex computing bio machines.^13^ Similarly as in lipids, natural functions of carbohydrates highlight the potential application of sugars in autonomous machines as structural, signaling moieties, or sources of energy. However, the utility of sugars as a functional part of NCAs is not as widespread as nucleic acids or proteins.

**Figure 1.**
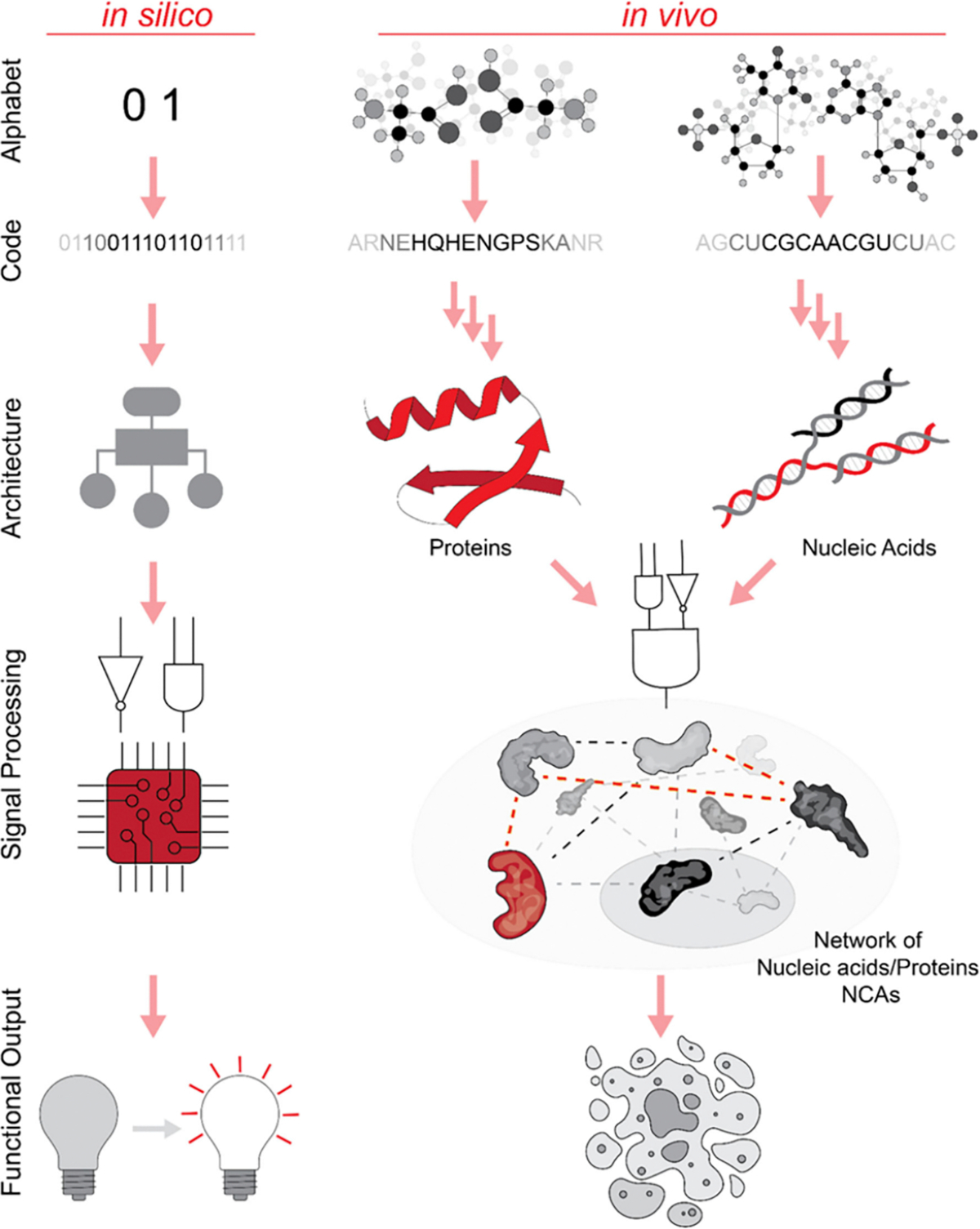
Schematic comparison of informational flow: from building blocks to functional output. In silico operations rely on machine language composed of two distinct abstract symbols: ON (1) and OFF (0), represented by physical states of low and high voltage. All information processing, interpretation, and execution are carried out by the central processing unit (CPU). In contrast, biological NCAs use two different chemical codes, either separately or in combination. The sequences of nucleic acids or amino acids result in higher-order architectures with unique autonomous functions that can be executed in vivo.

Nucleic acid NCAs, acting as memory-encoding polymers or standalone functional molecules, along with proteins, which serve as nucleic acid-encoded scaffolds or enzymes, represent the most suitable materials for designing conditionally responsive biomaterials (Figure 1, Table 1).^14^ Nanoparticles assembled from nucleic acids, proteins, or their combinations can be engineered as ready-to-go multifunctional nucleoprotein complexes, encoded by nucleic acid vectors, or enzymatically prepared from precursors.^15–18^ The primary challenge in the bottom-up development of smart NCAs lies in their design and selection. In nature, the evolution of subcellular nanomachines benefits from long time scales. However, the demand for novel therapeutics calls for more rapid strategies including the rational design and directed evolution of nucleic acids and proteins. The available methodologies span a broad spectrum, ranging from random strategies to rational design.^19,20^ The first approach relies on random mutagenesis of existing molecules to modify or acquire unique functions, while rational design modifies domains based on the understanding of their functionality. Rational design can also be used to evolve functional molecules *de novo*.^21^ In both cases, computational modeling of structural and physicochemical properties helps to predict the behavior of designed molecules.^22^ Although both methods have their advantages and disadvantages, the choice of method depends on the intended application of the nucleic acid or protein, often blurring the line between random and rational approaches.^23^ Rational design is frequently accompanied by directed evolution, which is a process of controlled selection to drive the evolution of nucleic acids or proteins with the desired properties. The core of the method comprises testing a large library of sequence variants, isolating the molecules with expected properties, and amplifying their templates for the next round. These repetitive steps allow enrichment of the best-performing molecules for their subsequent identification and further synthesis. Directed evolution can be performed on various levels *in vitro*, *ex vivo*, or *in vivo*.^24^

## NUCLEIC ACID NCAS

Nucleic acids are ideal building materials for nanotechnology due to their biocompatibility, programmability, and functional versatility. Their ability to form both canonical and noncanonical base pairs, especially in the case of RNA, results in a wide variety of structural motifs which can be adopted from natural sources or designed artificially for use as building blocks, similar to LEGO blocks.^25–35^ The programmability and dynamic responsiveness to environmental changes make RNA an attractive material for customized applications in biotechnology and personalized therapy. For example, noncoding RNAs (ncRNAs), perform a broad spectrum of functions to control and regulate gene expression at the transcriptional, posttranscriptional, and translational levels. They also guide nucleases or recombinases to genome-specific sites. Beyond gene expression regulation, many RNAs play vital roles in scaffolding, intracellular partitioning, and exerting antagonistic or agonistic effects on their protein targets.^36^ Recently, a novel group of cell surface-resident RNA molecules, which are covalently linked to sugars, has been suggested to function in immune cell interactions.^37,38^ All of these functions are encoded by the modular nature of nucleic acids, and when combined into single or multistranded molecules, their therapeutically synergistic logic-based behavior can be used to carry out computational logic circuits.^39,40^

The expanding field of therapeutic RNA nanotechnology encompasses a comprehensive understanding of RNA structure–function relationships and the roles that natural RNAs play in different diseases.^41–43^ This knowledge is crucial for addressing specific biomedical problems.^26^ It becomes possible to engineer synthetic pathways that mimic the orchestration of native regulatory biochemical processes by engineering functional RNA nanoparticles and NCAs that can communicate with one another and interact with cellular machinery to enhance the operation of therapeutic systems. Indeed, naturally occurring dynamic RNA molecules, such as metabolite- or cofactor-responsive riboswitches and ribozymes, illustrate the potential of RNA-based regulation. These examples inspire the design of artificial RNA NCAs tailored for specific therapeutic applications, improving precision and efficacy in disease treatments.^44–47^ When assembling various RNAs into more complex structures, however, it is essential to emphasize that such NCAs not only acquire different physicochemical properties and biological functions but also can change immunostimulatory responses. The composition (RNA vs DNA vs chemical analogs), shape, and size profoundly impact the nucleic acid nanoparticle’s interaction with the host’s immune system, as demonstrated by our team.^48–57^ The situation is further complicated by applied delivery carriers because various transfection agents alter the interaction of nucleic acid nanoparticles with cellular defense mechanisms.^58,59^

**Figure 2.**
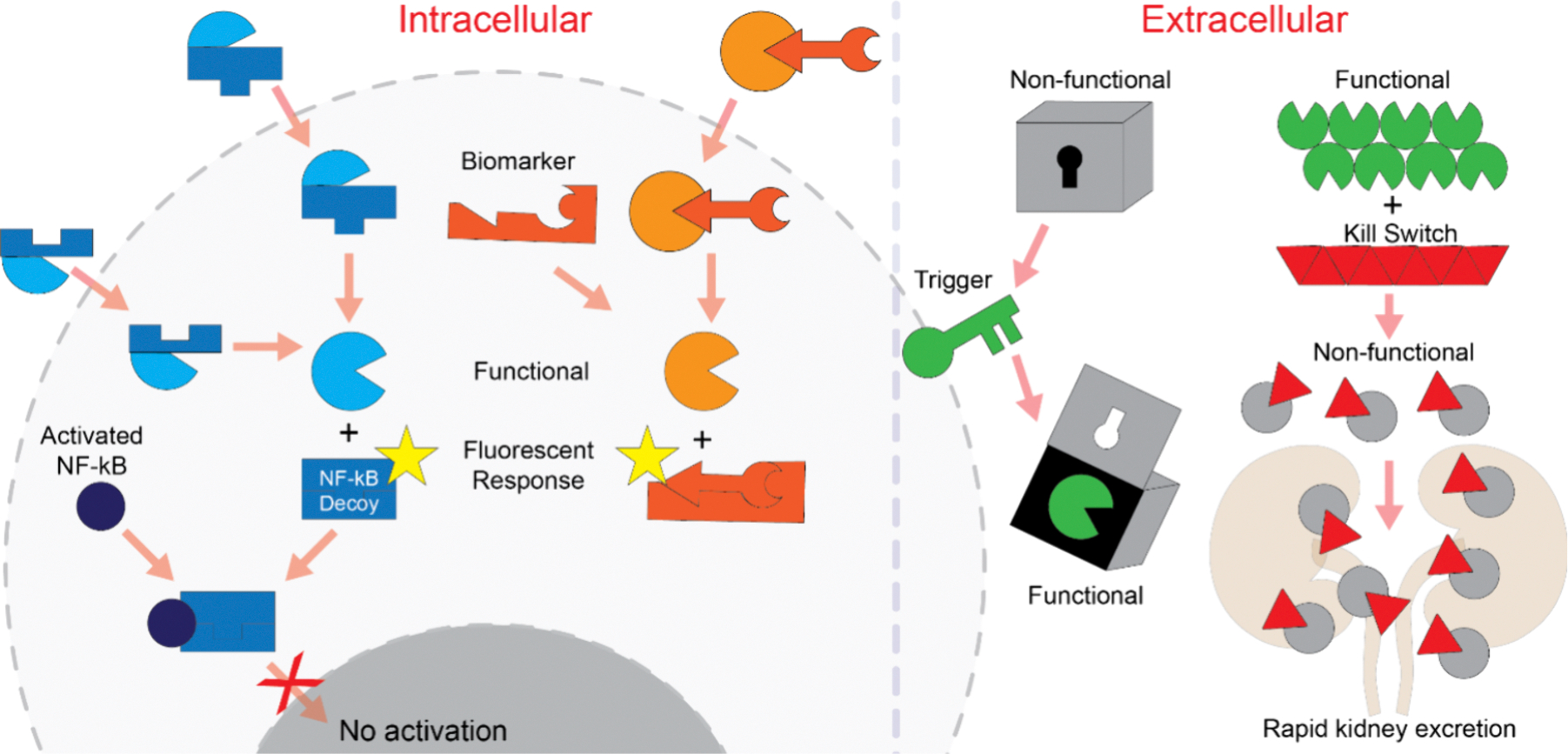
Schematics depicting basic strategies of functionality modulation. Two interdependent, individually nonactive split NCAs (blue) interact inside the cells, and strands are rearranged leading to functional molecules, e.g., transcription factor decoys, FRET signal, and RNAi inducers. Alternatively, NCA is activated upon sensing and binding endogenous triggers (orange). The presence of cell-specific receptors allows the binding of cognate aptamers displayed in complex DNA origami structures. Recognition of the receptors triggers a change in the NCA’s 3D shape, thus exposing therapeutic cargo. The kill switch on the other hand represents a reversible system to modulate blood coagulation. If necessary, the aptamer-fiber complex limits thrombin availability for a certain time, while injecting the kill switch releases thrombin from the bound state.

Many diseases frequently stem from the misregulation of gene expression or mutations.^60–62^ The differentially expressed genes can serve as potent biomarkers, distinguishing diseased cells from healthy tissues. This approach was utilized in one of the early attempts to build autonomous biomolecular computer that logically analyzed levels of disease related mRNA and in response released antisense ssDNA molecules.^63^ Several design strategies were then developed for nucleic acid NCAs to recognize specific molecular inputs (*e.g.,* overexpressed mRNAs or miRNAs) and link them to the specific output.^64–68^ The complementary interaction of nucleic acids ensures precise recognition that can promote intracellular activation of therapeutically relevant functionalities.^52,56,69,70^ The intracellular functionality could be conditionally induced through toehold interactions either by the presence of a specific cellular biomarker or when two complementary copies of dynamically interdependent nucleic acid NCAs are separately delivered into the same cell (Figure 2). Intracellular activity of NCAs in both cases is then turned on via thermodynamically driven reassociation and strand displacements that result in the lowest free energy active confirmations.^71,72^ The codelivery of two independent cognate functionalities can be stochastic; thus embedding multiple functionalities within one large hybrid particle can be a viable alternative.^73,74^ Several innovative approaches do not rely on toehold interactions to initiate intracellular reassociation, and their designing principles are straightforward without extensive computational assistance. The newly developed interdependent NCAs are designed by simply taking the reverse complements of the existing scaffolds and assembling them into the “anti-scaffolds”. As proof of concept, the interaction of complementary NCAs at physiological conditions results in shape-switching and the formation of various structures promoting diverse sets of functional platforms for *in vitro* transcription, Förster resonance energy transfer (FRET), fluorescent aptamers, intracellular gene silencing, and regulation of transcription factors and immunorecognition.^52,56^

In addition to the aforementioned strategies, a wide array of artificially designed simple, dynamic nucleic acid assemblies have been shown to respond to a broad spectrum of physicochemical stimuli (*e.g.,* light, temperature, and pH) or cognate molecules (*e.g.,* nucleic acids, proteins, and metabolites). Rationally designed DNA nanomachines have been used to carry out a rotary motion, switching from B- to Z-form DNA in solutions of increased ionic strength.^75^ DNA nanomachines sensing pH changes have been used to map acidity during endosome maturation, both in cell culture and in multicellular organisms.^76,77^ The same working principle has been applied to create two different particles for the simultaneous mapping of pH changes inside the same cell but in distinct endocytic pathways.^78^ The controllable photosensitive nanodevices are exemplified by photoresponsive DNAzymes with their activities being reversibly regulated by switching from visible to UV light.^79^ RNA thermometers are regulatory elements found within the untranslated regions (UTRs) of mRNAs, typically composed of hairpin structures or stem-loops that fold or unfold under a range of temperature conditions, forming at lower temperatures and melting at higher temperatures.^40,80,81^ The unfolding of these motifs allows ribosome binding sites to be exposed, thus initiating translation. These temperature-sensitive RNA thermometers have recently been explored as logic gates, with applications in cell-based and cell-free environments.^81–83^

DNA walkers and nanomotors are another large group of nanodevices capable of directional movement or motion based on strand displacement.^84,85^ Various strategies have been developed to promote the locomotion of DNA nanoparticles. In the simplest case, DNA walking is supported by stepwise strand displacements.^85^ Another DNA transporting system utilized a restriction enzyme to translocate an oligonucleotide cargo along the linear DNA trail.^86^ The direction of walking can be switched from forward to reverse by changing the nucleotide sequence of the fuel strands.^87^ Cleavage of RNA substrate by DNAzyme is a further possibility to power nanomotors to continuously conduct mechanical motion.^88^ The “molecular spider,” another innovative walker, moves in accordance with the prescriptive DNA origami landscapes.^89^ An interesting concept of a dynamic DNA “nanorobot” capable of cell-specific delivery and logical release of cargo in cell culture has been demonstrated based on the previous work of Andersen et al.^90,91^ The nanorobot was designed in the form of a hexagonal barrel split into two halves linked by single-stranded scaffold hinges. Two aptamers with corresponding partially complementary strands on the opposite domain kept the barrel in a locked state. The barrel was in the open state only when both aptamers simultaneously recognized target proteins and the lock duplexes dissociated. Similarly, an autonomous DNA robot constructed by DNA origami was programmed to seek and destroy tumors *in vivo*. Upon aptamer-based detection of tumor-associated nucleolin in endothelial cells, antinucleolin aptamers trigger a conformational change of the DNA nanobot that exposes thrombin inside out, which induces tumor intravascular thrombosis, leading to tumor necrosis and growth inhibition (Figure 2).^92^ DNA origami nanobots can dynamically interact with each other to control therapeutic molecules in a living animal.^93^

As another example of extracellularly acting nucleic acid NCAs, the application of “kill switches” introduced a concept of dynamic interactions among nucleic acid nanoparticles. Antithrombin aptamers have been extensively developed as safe and effective nonimmunogenic alternatives to traditional anticoagulants. However, suboptimal dosing observed in the Phase-I clinical trial of the antithrombin aptamer ARC183 resulted in its subsequent discontinuation.^94^ To overcome this, antithrombin aptamers were assembled in larger nanostructures with prolonged *in vivo* circulation and shown to efficiently prevent blood coagulation in two different animal models and human donors’ blood. This function was demonstrated to be reversible when kill switch constructs were coinjected. The preprogrammed interactions between the fibers and kill switches under physiological conditions led to the isothermal reassociation of strands, generating shorter duplexes. While the fibers, due to their size, bind to thrombin and are retained longer in the bloodstream, exhibiting a higher local concentration, the reconfigured shorter duplexes are functionally inert and rapidly excreted (Figure 2). This dynamic system highlights the potential of engineered nucleic acid NCAs for responsive and highly controllable therapeutic interventions.^95^

## PROTEIN-BASED NCAS

Designer “smart cells” controlled by an executable source code have been envisioned in the recent past. The essential central processing unit in this algorithm is protein-based NCAs, which integrate the fundamental input-process-output module within a single biomolecule,^96^ raising the question if we can regulate intracellular signal transduction pathways and remodel cell behavior using protein NCAs.

We demonstrated remote control of various signaling proteins by using special switches at allosteric sites, which changed the morphology of live cells.^97–99^ Building on this concept, we discovered that natural killer cells expressing an engineered blue-light-sensitive version of the septin-7 protein could move efficiently through constricted spaces in tumor spheroids. This suggests promising treatments for solid tumors surrounded by dense extracellular matrices.^100^ Overall, this emerging work highlights how NCAs can advance our understanding and enable tailored interventions in cellular homeostasis, signaling networks, and disease treatment.

**Figure 3.**
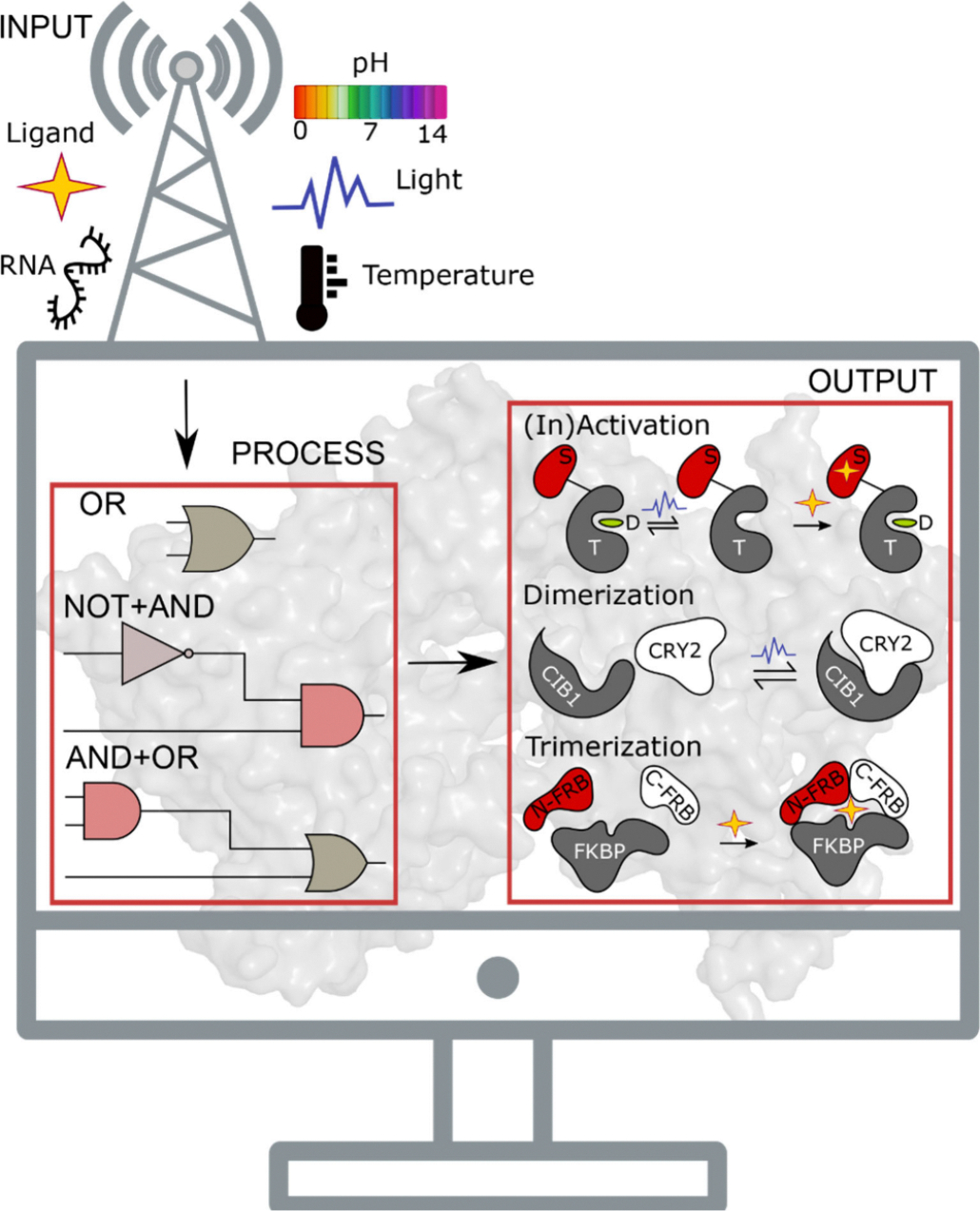
Protein-based NCAs are input-process-output modules. The core of an NCA is the target (T) protein which, when allosterically coupled to the sensor domain (S), yields a quantifiable output (activation/inactivation, dimerization, trimerization) in response to input cues, such as light, ligand, RNA, temperature or pH. The processing unit comprises of Boolean logic gates, featuring two-tiered regulation in the form of an OR gate^101^ or noncommutativity in the combinatorial NOT and AND gates.^102^ The output signal may be blue light induced rapid reversible inactivation of the target (T) protein,^101^ resulting in dissociation of its downstream effectors (D) or ligand induced irreversible activation.^97^ Light induced dimerization^103^ and ligand induced trimerization^104^ readouts, experimentally demonstrated in different target proteins, are schematized. FKBP: FK506 binding protein, FRB: FKBP12–rapamycin binding protein.

**Figure 4.**
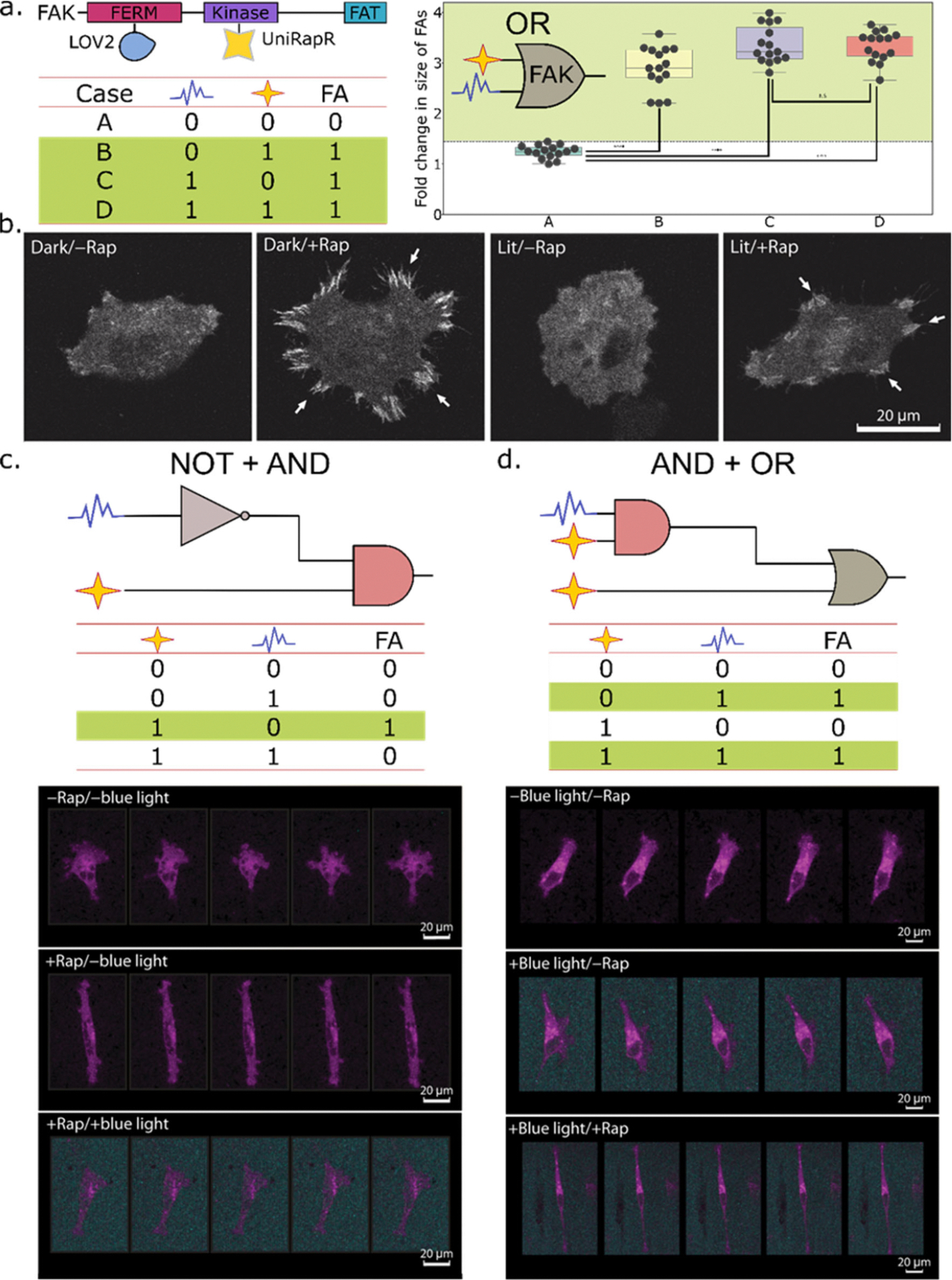
Two-tiered regulation and noncommutativity in circuits constructed using protein-based NCAs. (a) Domain organization of the target protein, focal adhesion kinase (FAK) and insertion sites of the sensor domains: LOV2 and uniRapR. An OR logic gate is fabricated using the chemogenetically activated uniRapR and the optogenetically activated LOV2 domains. The fold change in the size of focal adhesions (FA) in FAK−/− cells expressing the engineered FAK protein is significantly higher in the presence of either or both stimuli, i.e., rapamycin and blue light. Source data obtained from Vishweshwaraiah et al.^101^ (b) Focal adhesions form upon rapamycin addition, as seen by confocal imaging of fixed MDA-MB-231 cells transfected with “dark” or “lit” mutants of an engineered Src kinase containing the uniRapR and LOV2 sensor domains. Modified from Chen et al.^102^ (c) A combination of NOT and AND logic gates can describe the functioning of the chimeric Src kinase-based NCA, under dual regulation by blue light and ligand, when the ligand (rapamycin) is added first, (d) whereas a combination of AND and OR logic gates can explain its logical operations, when blue light is the first signal. Live-cell imaging shows changes in the orientation of the cell upon exposure to input cues. Modified from Chen et al.^102^ Source data for (a) are adapted with permission under a Creative Commons Attribution CC BY 4.0 International License from ref 101. Copyright 2021, The Author(s) (https://creativecommons.org/licenses/by/4.0/). Panels (b–c) reprinted (Adapted or Reprinted in part) with permission under a Creative Commons Attribution NonCommercial License 4.0 (CC BY-NC) from ref 102. Copyright 2023, The Authors, some rights reserved, exclusive licensee American Association for the Advancement of Science. No claim to original U.S. Government Works.

The functional unit of an NCA is the target protein, which displays distinct conformational states (analogous to Boolean logic gates) in response to input signals, producing quantifiable outputs (Figure 3). Sensor domains act as the interface for detecting and responding to cues such as ligands, light, pH, temperature, pressure, RNA, or other custom inputs. The sensitivity of these domains to the appropriate stimulus is typically exemplified in a large conformational change, which significantly alters the host protein function. Drug-activated switching domains, such as uniRapR,^97,98^ ER-LBD, and circularly permutated dihydrofolate reductase (cpDHFR)^105^ enable prolonged regulation of protein activity and accessibility to deep tissue compartments. On the other hand, the photoregulated light-oxygen voltage 2 (LOV2) plant origin domain,^106–108^ phytochrome B-PIF duo,^109^ cryptochrome 2-CIB1/CIBN pair,^103^ and a variety of other domains,^110–113^ governed by photonic cues, allow for noninvasive modulation of target protein activity while permitting high-resolution optogenetically driven spatial and temporal control. The combination of multiple activity modules has the potential to create more complex platforms featuring, for instance, two-tiered regulation; this concept has been demonstrated in the fabrication of an OR logic gate using uniRapR and LOV2 domains, to reconfigure the activity of focal adhesion kinase in live cells (Figure 4a).^101^ This work laid the foundation for the engineering of a rapamycin and blue light responsive-integrated circuit confined to a single protein, which combines the NOT and AND logic gates in a noncommutative manner to reversibly control Src kinase function *in vivo*, eventually impinging on cellular orientation and migration dynamics (Figure 4b).^102^ The introduction of noncommutativity in circuits holds promise given the potential to alter phenotypic outputs by different permutations of a modest number of regulatory domains (Figure 4c–d).

Harnessing inherent allostery in target proteins is a recurrent theme in the rational design of protein NCAs^99,114^ The installation of small regulatory domains (~15 kDa) involves the generation of chimeric constructs that leverage allosteric regulation,^115,116^ direct modulation of protein activity through steric interference afforded by the sensor domains,^117,118^ or signal-inducible reconstitution of split proteins,^104,119–121^ where functional modulators must have a 7–12 Å end-to-end distance.^119^ Allosteric coupling transfers the sensitivity of insertable sensor domains in NCAs to external cues, typically manifested as an optogenetic or chemogenetic signal-mediated change in structural order (ordered-to-disordered (or *vice versa*) transition^122,123^), to the active site of the response unit. Using experimental techniques, such as site-directed mutagenesis,^124^ in conjunction with molecular dynamics simulations and biochemical screens against compound libraries and X-ray crystallography^125,126^ to identify the allosteric network for target proteins, is time-intensive. Wang et al. proposed network-based protein structure-guided computational algorithms, Ohm,^127^ founded on the principles of perturbation-propagation, that readily maps allosteric sites and provides inter-residue allosteric coupling information^128,129^ crucial to the insertion of the regulatable scaffolds.^130^

Stable multidomain proteins, which can withstand changes in pH, temperature, or intermolecular interactions, offer a versatile, customizable platform for integrating diverse functional modulators. Several protein families such as kinases,^97,98,118,131–136^ metabolic enzymes,^106^ motor proteins,^137^ regulatory proteins,^97,138–141^ inteins,^142^ and nanobodies^105,143^ have been allosterically regulated using a wide spectrum of photoswitchable or ligand-controlled sensor domains. The specific response is manifested in the multitude of functions that proteins can perform, including but not limited to biocatalysis, change in subcellular localization,^144^ oligomerization, and protein–ligand interactions (Table 2).

In contrast to strategies aimed at cellular reprogramming founded on protein expression regulation during transcription or translation or modulation of protein–protein interactions and proteolysis at the post-translational level, NCAs offer great advantages, including robustness, DNA code compaction, enhanced metabolic efficiency, reduced expression variability, faster response times, and higher spatiotemporal precision. Additionally, NCAs coupled with light-sensitive functional modules present reversible photoswitchable protein inactivation. However, NCAs with sensor domains should operate covertly, only to activate and transmit the response to the target protein in the presence of the appropriate stimulus, a feature that accounts for the specificity of the response. Furthermore, the endogenous protein function or intracellular interaction network must not be affected upon insertion of each sensing domain. For experimental consistency, NCAs should be temporarily transfected or embedded in the cellular genetic material in the form of a stable cell line.^96^

Although NCAs with controllable logic have been crafted, the challenge lies in simultaneously engineering multiple proteins to build *in vivo* “intelligence” reporting on protein function, structural changes, and conformational dynamics. This surveillance network could potentially analyze, manipulate, and drive cellular interactions, and consequently steer cellular behavior with far-reaching impacts on precision medicine and therapeutics. Key milestones in bioprogramming may be achieved with the reconfiguration of the allosteric framework in proteins and the expansion of the catalog of input signals. The existing protein-based NCAs are on–off switches. The methodical design of a continuous modulable response generator, like a rheostat, would allow for streamlined and refined control. The conceptualization and realization of innovative design principles and rational combination of chemically distinct biomolecules in the fabrication of NCAs can chart unexplored avenues in biocomputation.

## COMBINATION OF CHEMICALLY DIFFERENT NCAS

The integration of rationally designed proteins and nucleic acids into cohesive NCAs leverages the unique advantages of both biomolecule types.^147,148^ Proteins, with their diverse structural motifs and functions, enhance the programmable nature of nucleic acids. As detailed in the previous section, proteins have been engineered to respond to specific stimuli, such as light or drugs, and perform complex computational operations within living cells. These protein-based NCAs can integrate sensor domains that trigger changes in cellular behavior without requiring gene expression or suppression, enabling rapid and efficient molecular computation.^96,101,102^ Alternatively, nucleic acids offer exceptional programmability and molecular recognition. Utilizing canonical and noncanonical Watson–Crick base pairing, DNA and RNA can be precisely engineered to form complex nanostructures, creating three-dimensional architectures with defined properties. These NCAs can be engaged in logical operations to act as molecular switches or serve as dynamic and stimuli-responsive scaffolds for other functional elements.^48,149–151^ To create hybrid DNA-RNA-protein NCAs, the precise design and functionalization of both components is essential. Engineering proteins interacts with RNA molecules in a controlled and predictable manner and can facilitate complex molecular computations. For example, DNA origami structures can be precisely programmed to position engineered proteins.^152^ Conversely, proteins can be designed to interact with specific DNA or RNA sequences, facilitating the dynamic regulation and control of nucleic acid-based nanodevices.

In the past decade, RNA-guided nucleases such as the CRISPR-Cas systems, have shown their enormous potential and versatile interactions within the genome to function not only as nucleases or nickases, but also as transcriptional activators, repressors, epigenetic modulators, or modulators of genomic architecture.^153^ 2023 was a milestone year for the field of gene therapy, as the first CRISPR-Cas-based therapy was approved in the US, followed by the EU in 2024 (Press Release).^154,155^ The guide RNA has been shown to be amenable to functional alterations including conditional activation by ssDNA or ssRNA oligonucleotides.^156–158^ A recently described “bridge RNA” consisting of two RNA loops encoded by a transposable element (IS110) that also encodes recombinase can be independently programmed to target specific genome sequences. This enables the combination and matching of any two cleaved desired DNA sequences. The bridge RNA recombination system derived from IS110 expands the scope of nucleic acid-guided technologies and provides a mechanism for fundamental DNA rearrangements, such as insertions, excisions, and inversions.^159^

Recent studies have proposed the development of a universal interface that bridges enzymatic and DNA computing systems. The selected enzymes process small molecules, producing NADH, which triggers the release of an oligonucleotide for DNA computation. This interface allows for the flexible integration of various components, enabling a wide range of biocomputing applications.^160^ The precise engineering of DNA–protein hybrid nanostructures has created intricate molecular architectures with controlled spatial arrangements and orientations, providing opportunities for designing functional materials, biosensors, and therapeutic agents.^161,162^ Furthermore, biomolecule-based Boolean logic gate systems, constructed from nucleic acids and/or proteins, operate as fundamental building blocks for complex computational circuits, performing logical operations such as AND, OR, and NOT.^163,164^ These advancements have significantly expanded the capabilities of biomolecular logic gates, enabling the creation of more intricate circuits, faster computational speeds, and potential applications in fields like diagnostics, therapeutics, and environmental monitoring.^165^

RNA-protein (RNP) complexes represent another exciting frontier in biomaterials and nanotechnology. Combining the structural versatility of RNA with the functional diversity of proteins, RNPs can be precisely engineered for a wide range of applications, including targeted drug delivery, biosensing, and enzyme catalysis.^150,166,167^ Additionally, expanding the genetic code to incorporate noncanonical amino acids (ncAAs) has prospects for protein engineering. The ncAAs can be used to probe biological mechanisms, enhance enzyme activity, and introduce unique catalytic functions into protein-active sites. The expanded amino acid repertoire enables the creation of enzymes with unique properties and functionalities, making ncAA incorporation a valuable tool for biocatalysis and synthetic biology research.^168,169^ Furthermore, synthetic analogs such as peptide nucleic acids (PNAs) and incorporation of unnatural base pairs into nucleic acids have significantly broadened the structural and functional capabilities of nanocomputing agents. PNAs, which combine features of both proteins and nucleic acids, show a high affinity for DNA and RNA targets while providing enhanced chemical stability and resistance to enzymatic degradation. This hybrid approach allows for the creation of more robust and versatile nanocomputing systems that can operate under various conditions, both *in vitro* and *in vivo*.^170–173^

A key aspect of this integration is the accurate prediction of RNA and protein 3D structures and functions, which play a vital role in the successful design of NCAs. Advanced computational methods, such as those implemented in RNA 3D structure prediction tools,^174^ enable the detailed modeling of RNA folding and conformation, such as iFoldRNA,^175–177^ SimRNA,^178^ Dragnent Assembly of RNA with Full-Atom Refinement (FARFAR),^179^ 3dRNA,^180–182^ RNAcomposor,^183^ Vfold,^184^ RhoFold,^185^ DeepFoldRNA,^186^ and AlphaFold 3,.^187^ These prediction methods facilitate the modeling of accurate RNA structures that can be attached to proteins in a way that ensures stable and functional hybrid NCAs.^188,189^

The integration of proteins and nucleic acids as NCAs represents a significant advancement in biocomputing. This approach combines the precision of nucleic acids with the functionality of proteins, creating highly sophisticated and versatile nanocomputing agents that leverage the strengths of both proteins and nucleic acids and are capable of performing complex biological and computational tasks. While 3D predictive modeling allows for insights into structural complexity based on the known building blocks of proteins and nucleic acids, other predictive tools can be used to perform design based on experimental results. For instance, an online platform called *Artificial Immune Cells (AI-cell)* has been introduced to predict the immune activities of engineered multistranded nucleic acid nanoparticles in human blood cells.^49^ This tool, based on the transformer architecture, is publicly available at https://aicell.ncats.io/ and can help guide the design of nucleic acid NCAs intended to communicate with the human immune system. Experimental validation within a relevant biological context is crucial to provide a comprehensive overview of the function of NCAs, especially those driven by environmental cues or intended for intracellular applications.

## HIGH-THROUGHPUT EVALUATION AND IDENTIFICATION OF THERAPEUTIC CANDIDATES IN CELLS

The rational design of autonomous therapeutics offers the potential to account for safety, efficacy, and pharmacokinetics early in the design stages of drug development, all of which are continuous hurdles for drug candidates entering the preclinical pipeline. This strategy is made possible by a systematic feedback loop in which experimental analysis can be used to improve a design hypothesis.^190^ Furthermore, experimental data can be used to train artificial intelligence, resulting in machine learning models, directing the evolution of predefined drug discovery.^191^ For efficient drug design, it is crucial that experimental data be generated in human-relevant preclinical models that can mimic the functional complexity of the biological environment. Data from animal models consistently fails to accurately predict the results of human clinical trials,^192^ which can result in therapeutic candidates and design principles that are suboptimal for patient use if incorporated into the feedback loop. As an alternative strategy, innovative methodologies that introduce more relevant *in silico* and *in vitro* models are of great interest to improve drug discovery, development, and predictability, with their usefulness highlighted by the redefinition of nonclinical testing in the recently passed FDA Modernization Act 2.0.^193,194^

Complex *in vitro* models are multicellular cultures, often with a three-dimensional structure and under dynamic fluid flow, which can imitate the architectures and functions of human biology.^195,196^ These models can be engineered with increasing precision and can utilize primary patient-derived material to establish a baseline tissue function for extended periods in culture.^197^ These features better support physiological relevance over two-dimensional cell studies, as indicated by gene expression and the ability to capture functions such as metabolism, transport and barrier integrity, immune cell circulation, recruitment, mechanical cues, and shear stress, thereby establishing a more relevant biological environment for the evaluation of drug candidates.^198–202^ These functional *in vitro* tissues can also be applied beyond healthy phenotypes to model disease conditions which may be important for evaluating therapeutic efficacy.^197^ For example, it has been shown recently that nanoparticles introduced into melanoma-on-a-chip had greater accumulation and retention as a result of nanoparticle size and transferrin receptor targeting. Optimal nanoparticle characteristics from work in spheroids were also confirmed within a murine xenograft, demonstrating the utility of alternative models.^203^ By recapitulation of environmental or disease triggers, complex *in vitro* models can be used to evaluate functional NCAs in a more relevant cellular environment. For instance, key microRNA biomarkers for prostate cancer were observed to be upregulated in clinical samples and in 3D cultures relative to 2D cultures.^204^

Incorporating complex *in vitro* models into rational design has the additional potential to benefit time, scalability, and thus the cost of overall development due to the ability for high-throughput screening of hundreds to thousands of candidates.^205–207^ With this approach, large compound libraries can be screened for readouts of interest to identify “hits” that further enrich design principles.^208^ This could be particularly advantageous for autonomous therapeutics that are highly modular with recurring motifs that could be correlated with experimental readouts.

## DELIVERY OF NCAS *IN VIVO*

Specific delivery of therapeutic agents *in vivo* is generally the bottleneck for successful translation of many therapies into the clinic. Therapy of some cells, mostly from hematopoietic systems, can bypass the obstacle of *in vivo* targeting by their removal and subsequent *ex vivo* treatment followed by the reintroduction of cells to patient’s body. However, most cells/tissues are not accessible for *ex vivo* therapy. Therefore, cell specific delivery to diseased cells in safe and efficient dose poses a significant objection.^209^ Smart NCAs can be designed to sense and activate their functionality only inside cells with specified disease-associated molecular signatures, thus minimizing the necessity for targeted delivery. However, this option may require the application of higher doses to compensate for uptake by nonrecipient tissues. The NCAs can be delivered in form of (i) templates where active components such as RNA and/or proteins are produced in host cells (*e.g.,* DNA templates for nuclease and guiding RNA);^210^ (ii) ready-to-go arrangements (*e.g.,* nuclease and guiding RNA as RNP complex),^211^ or (iii) combined setting-template and final molecule (*e.g.,* mRNA for nuclease and sgRNAs).^212^ The successful transfer of NCAs to the site of their action is influenced by multiple biological factors. Upon administration, the cargo must be protected against degradation and immunorecognition while still binding to cell specific receptors and accessing the cytoplasm for cargo release. As exemplified by current efforts for *in vivo* delivery of gene editing tools, viruses, virus-like particles (VLPs), and lipid nanoparticles (LNPs) are foremost in state-of-the-art delivery systems.^209,213^ Virus vectors capitalize on their naturally evolved abilities to effectively infect the cells. Three main types of viruses were deployed as vectors of gene editing tools: Adeno-associated virus, Lentivirus, and Adenovirus. Differences in their genome capacity, immunogenicity, retargeting feasibility (incorporation of receptor specific molecules), genotoxicity and duration of activity influence the choice of viral vector.^214^ LNPs are synthetic carriers composed of several types of lipids. Variability in composition of LNPs offers different pharmacokinetic properties and several formulations were already approved by US FDA.^215^ Intravenous administration of LNPs usually ends up in hepatocytes due to coating by blood lipoproteins and their uptake by hepatocyte receptors. Cell specific nonliver delivery of LNPs (*e.g*., through conjugation with antibodies) will significantly broaden the application of LNPs.^215^ Without targeting ligands, the application of LNPs is restricted for local administration (muscles, retina, inner ear, *etc*.). The VLPs are promising retrovirus derived carriers for delivering NCAs due to their noninfectious nature and ability to package mRNAs, proteins, or RNPs, including or instead of viral genetic material. VLPs are taking advantage of natural viruses’ properties-structurally ideal for encapsulating diverse cargos and allowing separate modulation of cargo packaging via capsid proteins while customization of cell targeting through envelope glycoproteins.^216^ In summary, exploiting naturally produced carriers (viruses) is more effective than utilizing synthetic approaches (VLPs); however, the former is more vulnerable to stochastic outcomes. For consistency in performance, protein-based NCAs must be genetically encoded. Transient transfection or generation of stable cell lines is the method of choice for introduction into the cells. Such methods offer simplicity and reproducibility in the engineered logic circuits.^96^

## CURRENT STATE OF AFFAIRS: SCALE, THERAPEUTIC APPLICATIONS AND LIMITATIONS

With the essential toolkits at our disposal, we are poised to achieve a breakthrough in terms of the development of a biomolecular scale programming language. The following designs are some illustrative examples of the scale of computation that has been currently accomplished. In a pioneering study, protein-based nanocomputing agents which can function as multiplexors, were fabricated.^97^ This work provides a proof-of-concept prototype demonstrating that the functions of multiple proteins can be modulated simultaneously in living cells in the context of light-controlled activation and inhibition. It also lays the groundwork to investigate how light-induced activation of a single engineered protein is altered through the downstream inactivation of another protein. In another study, Boolean logical operations were combined to create combinatorial logic circuits which operate in a noncommutative manner (Figure 4, Table 2),^102^ with potential applications in biomechanical engineering and medicine. In many studies, control of cellular behavior was achieved using a single nanocomputing agent. A recently published report by Chen et al., demonstrates improved penetration of immune cells expressing an engineered photoactivatable variant of septin-7, into solid tumors.^100^ It is thus readily conceivable that the development of precision therapeutics using such NCAs is steadily taking shape with significant advances in biomolecular design and engineering.

NCAs exhibit distinct operational characteristics compared to traditional computers (Figure 1), particularly in terms of their core performance metrics. While NCAs can process multiple inputs simultaneously through protein-based responses to physical and chemical stimuli, their computational speed generally is considerably lower than that of electronic computers due to their reliance on atomic-scale interactions.^217^ In terms of scalability, NCAs show promise in incorporating multiple sensor domains and producing diverse outputs based on input combinations. However, current technical limitations constrain this scalability. Reliability remains a significant challenge, as demonstrated by research highlighting the need for precise environmental conditions to ensure consistent protein responses to stimuli such as light and rapamycin. The system’s computational power is limited by its reliance on simple input–output relationships, the requirement for precise molecular-level control, and the difficulty of maintaining consistent performance across varying cellular environments.

NCAs are currently limited to two-input logic gates. Expanding the repertoire of input signals is the key to the generation of multiple-input logic circuits. Conditional activation in the presence of input signals such as RNA, pH, temperature, and metals would help create contextual biomolecular sensors. Chemically distinct biomolecules such as lipids, macrocycles, and metabolites may be employed in the architecture of NCAs. The testing of NCAs is by far, restricted to *in vitro* biochemical assays, and cell culture systems. Clinical testing of NCAs in whole organisms is a future endeavor. While conceptually viable, the remodeling of allosteric networks in proteins is yet to be established; an idea that would inevitably lead to a giant leap in bioprogramming. Despite these limitations, NCAs hold potential for specialized applications, especially in biological computing where traditional electronic systems are ineffective.^218^

## AUTONOMOUS FUTURE

**Figure 5.**
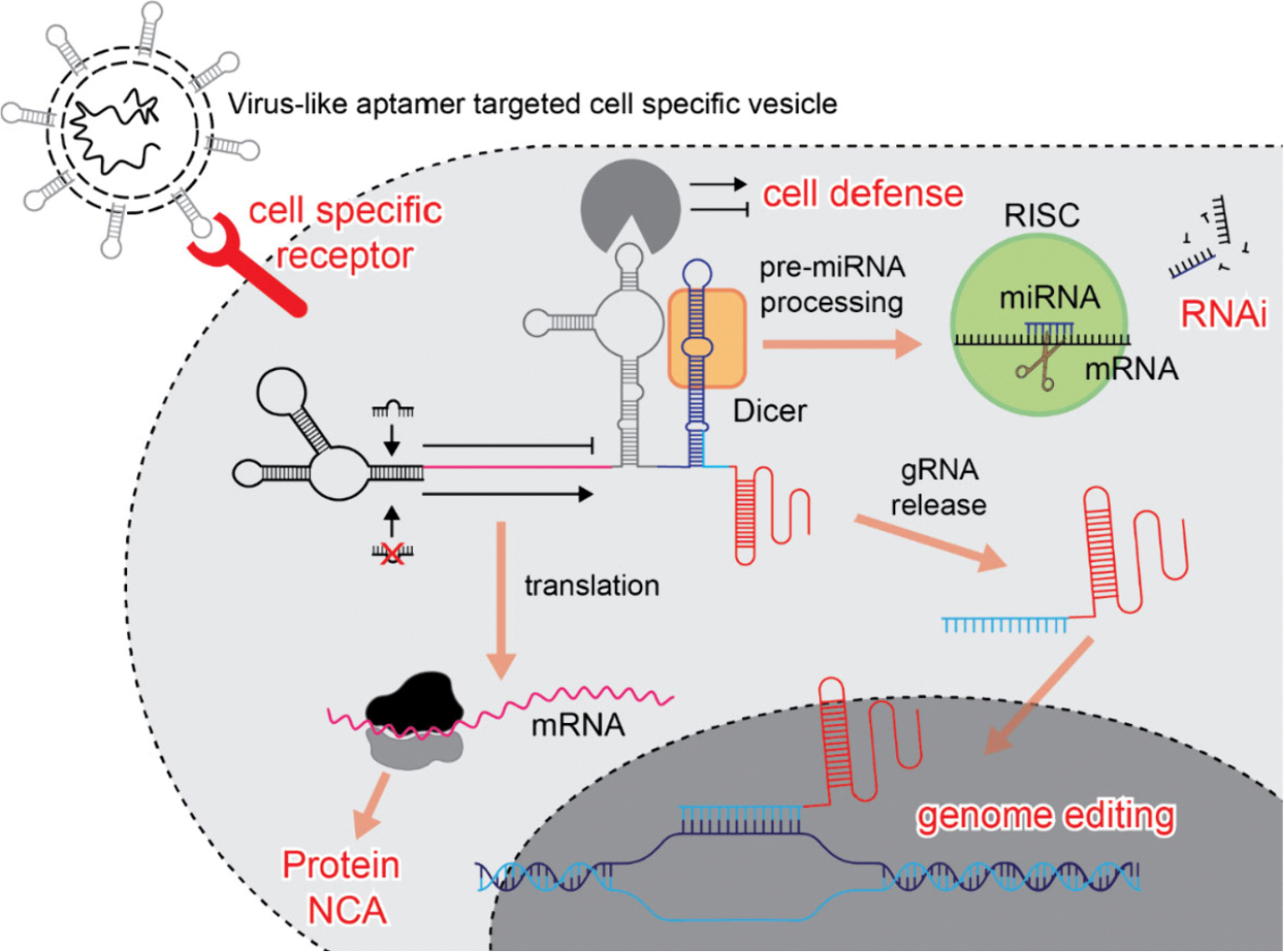
Hypothetical activity of NCA-virus-like aptamer-targeted cell specific vesicle. After cell specific binding mediated through DNA/RNA aptamer-receptor, the vesicle is internalized (not shown) and based on the presence or absence of endogenous molecular triggers modulate activities of encoded NCAs. Depicted NCA carry on CRISPR-Cas protein and guide RNA (gRNA) that result in genome editing controlled by RNAi. Embedded aptamer in central region can stimulate or inhibit cellular immune response to NCA. Distal part of the RNA contains pre-miRNA fused to gRNA. The pre-miRNA can be processed by Dicer and subsequently loaded to RISC complex and regulate expression of targeted gene. Dicer processing of pre-miRNA also liberates gRNA, which could be loaded into CRISPR-Cas protein if expressed. Suggested systems could be enriched with other functional moieties or advanced logic interactions with cellular components.

Smart, nature-derived, and *de novo* artificially evolved nucleic acids/proteins or combinations of both in nucleoprotein complexes are steadily populating the field of experimental biomedicine. Currently, nucleic acids are more straightforward for design, manipulation, and synthesis compared to proteins. Nevertheless, protein-based NCAs integrate input cues, processing, and response in a single molecular entity, resulting in greater robustness, code compaction, high metabolic efficiency, reduced expression variability, and rapid turnaround times. Ensuring the specificity of endogenous triggers (transcripts, proteins, or metabolites) will require extensive mapping of their presence in different tissues under physiological and pathogenic conditions. Alternatively, while using multiple interdependent molecules *in vivo* or exogenous molecular stimuli (activator or deactivator), all units must be delivered in the target tissue in the same or similar quantity. This condition is challenging due to the uneven distribution of multiple interdependent molecules within the body after entering systemic circulation. Therefore, the highest level of specificity and autonomous functionality will be achieved with the development of robust cell-specific targeted delivery methods to distinct tissues. This will result in reduced drug dosages with a higher probability of both moieties achieving effective levels in targeted cells rather than split allocation of multiple moieties. The feasibility of multiple split functionalities has been successfully tested in cell cultures, but further studies are needed to determine efficient modes of drug delivery before testing *in vivo*. Redundancy and parallel signaling pathways will also require combinatorial logic control and strategic design to achieve synergistic therapeutic outcomes (Figure 5). This is exemplified by cooperative targeting in cancer therapy to overcome intratumor heterogeneity and plasticity, making tumor cells resistant to the therapeutic targeting of common molecular pathways.^219^

The continued development of these future biomedicines through either rational design or random mutagenesis strategies is expected to yield combinatorial libraries of potential candidates. Experimental results that give feedback on the design process will be beneficial for evaluating, isolating, and generating the next iterations of the nanocomputing agents. To this end, high-throughput evaluation of the efficacy of NCAs in relevant cell culture models and predictive models based on these results offer avenues for the development of such therapeutic modalities.^220^

The logical circuits designed in laboratories, to date, mark only the beginning of “bioprogramming”, demonstrating that biomolecular function can be regulated using logic operations embedded in a hybrid NCAs. Such biological nanocomputers are expected to have far-reaching impacts in the understanding of biological processes and disease conditions and in the development of precision medicine, disease diagnostic devices, drug delivery systems, and reprogramming of cell signaling. Further, they may have potential biotechnological applications, which are not related to biological systems. In addition, NCAs may be designed to adapt under the influence of innate evolutionary pressure, paving the way for experimental therapeutics.

Incorporating machine learning (ML) could significantly enhance the design and functionality of the NCAs. By leveraging advanced predictive models like AlphaFold3,^221^ which extends AlphaFold’s capabilities beyond protein structure prediction to include RNA, DNA, and even small molecules, researchers can more accurately model the structures and optimize the design of NCAs. This enables precise forecasting of the behavior and interactions of NCAs, such as the DNA-based logic gates, reducing the risk of mismatches or off-target effects and improving the robustness and adaptability of NCAs in complex biological environments. AI algorithms can also help identify and correct potential mismatches or off-target effects, enabling the construction of more reliable NCAs that operate seamlessly within variable biological environments. Additionally, AI can help researchers tailor these systems to specific, adaptive responses that increase their robustness and efficacy for biomedical applications.^222^ By using AI- and ML-assisted strategies, researchers can rapidly identify nucleic acid or protein variants with enhanced responsiveness, stability, or specificity, significantly improving NCA outcomes in a fraction of the time required by traditional methods.^223^ This accelerated approach allows for the efficient refinement of NCA properties, supporting the development of highly adaptive, programmable biomaterials with applications in biotechnology and biomedicine.

In the near future, we can anticipate advances in the development of precise targeting and improved sensitivity and specificity of autonomous NCAs. However, on the horizon, currently speculative yet equally intriguing questions arise: How many functions can nucleic acids and proteins embody while maintaining communications and intramolecular interactions within the cellular milieu? Can we rationally design constructs for extended lifetimes within the cell that will perpetually sense and respond to pathogenic states? How can viral RNA genomes that exhibit high functional density and versatility^224–227^ continue to be adapted^228^ for prospective clinical use? Though currently fictional, is it possible to design and develop safe, self-evolving, autonomous therapeutics?

## Figures and Tables

**Table 1.** Comparison of Key Advantages and Challenges for Nucleic and Protein Based NCAs

Nucleic acid NCAs	Protein NCAs	Nucleic acid–Protein NCAs
	**Advantages**	
• Programmability • Biocompatibility • Self-assembly • Dynamic behavior • Molecular sensing • Scalability and modularity • Inherent catalytic activity • Low energy requirements • High information density	• NCAs do not necessarily control protein activity by controlling localization or function by controlling signaling cascades using multiple components, but rather by generation of a chimeric target protein, with modifications away from the active site, thus resulting in significant “code compaction” • Optogenetic signals allow reversible and rapid activation of target • Minimal response times • Low metabolic cost • No loss of signal via diffusion • Heterogeneity in expression levels is minimal, thus making the code reliable and reproducible • Structural diversity and functionality	Robustness • Enhanced metabolic efficiency • Reduced expression variability • Faster response times • Higher spatiotemporal precision
	**Challenges**	
• Structural and chemical instability of naked, nonmodffied nucleic acids • Complex folding pathways • Functional scalability and complexity • Environmental sensitivity • Inefficient cellular uptake and localization • Immunorecognition and uncontrolled immune responses • Off-target effects	• Availability of conclusive biochemical or *in vivo* assays for protein activity • Insertion of sensor domains is feasible only for stable target proteins. • Target proteins should ideally have “tight” surface-exposed loops connecting structured domains, for sensor domain insertion. This may not be the case for transmembrane and intrinsically disordered proteins. • It is important to consider phototoxicity and accessibility of target proteins to light, for efficient optogenetic control • Complex design and engineering • High production costs • Limited shelf stability	• Design and selection • Naturally occurring motifs take longer time scales
	**Underlying mechanisms**	
• Shape shifting • Complementary base pairing • Electrostatic interactions • Toehold-mediated strand displacement • DNA/RNA–protein interactions	• Conformational changes • Signal transduction • Enzyme kinetics and catalysis • Post-translational modifications • Protein–DNA/RNA interactions • Optogenetic and chemogenetic control • Protein-based logic gates • Scaffold and compartmentalization	• Combination of nucleic acid and protein NCAs
	**Representative applications**	
• Regulations of gene expression • Guiding of nucleases • Protein/small molecule binding • Scaffolding • Logic gates • Signal amplification mechanism • Environmental responsiveness	• Protein regulation • Study of physiological processes • Study of disease phenotypes • Development of experimental therapeutics • Potential biotechnological applications	• Conditional CRISPR-Cas therapies • Conditional epigenetic control • Responsive drug Activation • Programmable agents in infection, cancer therapy etc.

**Table 2.** Salient Features of Protein-Based NCAs

NCA type	Design	Logic operation	Mechanism	Cellular phenotype or Application
Protein-based	Insertion of iFKBP destabilizes and inactivates the target protein, which is activated upon rapamycin and FRB binding.^118,131^	Single chemogenetic input signal, rapamycin	Allosteric site modulation leveraging iFKBP-FRB interaction (ligand inducible dimerization system). Example of light-inducible dimerization system includes CRY2-CIB1 (Figure 3)^103^	Ligand-induced target protein activation and downstream signaling. Coexpression of FRB introduces cell to cell heterogeneity in expression and activation levels.
Protein-based	uniRapR domain (fused single chain iFKBP-FRB) insertion inactivates the target protein, which is activated upon interaction with rapamycin or its analogs.^97,98^	Single chemogenetic input signal, rapamycin	Allosteric site modulation of target protein	Ligand-induced target protein activation and downstream signaling, bypassing requirement of FRB coexpression.
Protein-based	RapR-TAP: iFKBP is inserted in the target protein, while FRB is tagged with a specific downstream binding partner or subcellular targeting sequence.^119,145^	Single chemogenetic input signal, rapamycin	Allosteric site modulation leveraging iFKBP-FRB interaction	Ligand-induced target protein activation only in specific subcellular compartments or only upon interaction with specific binding partners.
Protein-based	Photoactivable Rac1 GTPase: The C terminus of LOV2 is fused to the N terminus of Rac1 Insertion of LOV2 in surface-exposed “tight” loops of Src kinase and Rho family GEF and GTPase.^97^	Single optogenetic input signal Single optogenetic input signal (NOT)	In dark conditions, LOV2 sterically hinders the Rac1 active site, which is accessible with the unwinding of the j*α* helix in presence of light. Light-induced allosteric modulation. Inactivation of target proteins in the lit condition.	Pivotal function of Rac in cell motility elucidated using this engineered construct.^141^ Role of Rac in learning and memory in mice studied.^146^ *In vivo* control of protein activity
Split protein-based multimerizationsystem	The target protein, which is split into two or more lobes, is assembled. Potential sites of splitting can be computationally identified using the SPELL approach.^121^	Single optogenetic or chemogenetic input signal	Exploiting chemical (e.g., iFKBP-FRB-rapamycin)^120^ or light (e.g., CRY2–CIBl) induced multimerization systems ^103^ (Figure 3).	Protein regulation
Protein-based	Photoactivable LOV2 and rapamycin-responsive uniRapR sensor domains inserted in the FERM and kinase domains of FAK respectively.^101^	OR	Allosteric site modulation	Change in cellular membrane dynamics, especially size and number of focal adhesions
Protein-based	Rapamycin-inducible uniRapR and blue light-sensitive LOV2 domain inserted into human Src kinase.^102^	NOT+AND (The protein device behaves as a noncommutative combinatorial logic gate; Figure 4) AND+OR (Figure 4)	Allosteric site modulation	Reversible control of cell migration dynamics and orientation. Potential applications in tissue engineering and regenerative medicine.
Protein-based	Photoactivable LOV2 domain inserted in the target protein, septin-7.^100^	NOT	Allosteric site modulation	Improved immune cell penetration into solid tumors
